# Control interno de la calidad y garantía externa de la calidad: un gran pasado abre el camino a un brillante futuro

**DOI:** 10.1515/almed-2022-0080

**Published:** 2022-08-23

**Authors:** Mario Plebani

**Affiliations:** Universidad de Padua, Padova, Italia

**Keywords:** control externo de la calidad, control interno de la calidad, calidad, error total tolerable, incertidumbre del valor

En este número de la revista, publicamos dos artículos de referencia sobre la historia, evolución y papel fundamental de las principales herramientas utilizadas en los laboratorios clínicos para la evaluación, revisión y mejora de los resultados analíticos, que son el control interno de la calidad (IQC) y la garantía externa de la calidad (EQA, por sus siglas en inglés) [1, 2].

La lectura de estos artículos será bienvenida no solo por los lectores de la revista, sino por toda la comunidad de profesionales de laboratorio, dado que consideramos que es un buen momento para actualizar nuestros conocimientos sobre IQC y EQA y, lo que es más importante, determinar si estas herramientas se siguen empleando más “por tradición” que porque realmente sean eficaces a la hora de mejorar la calidad analítica y fiabilidad de los resultados analíticos. Los autores, acertadamente, definen el propósito de la IQC como “revisar el proceso de evaluación, con el fin de evitar que se genere información errónea relativa al estado clínico del paciente” [1], y resumieron las raíces de la IQC en la siguiente afirmación: “el modelo más antiguo, desarrollado en los años 50, se basaba en un criterio estadístico, mediante el cual se calculaba la media de un número de resultados para un único analito en la misma muestra de control. Esto se complementaba con el uso de una gráfica de control, que representaba los resultados de las muestras de control en el eje Y con respecto al tiempo o día en el eje X”. A continuación, pasan a describir la mejora sustancial que conllevó el uso del algoritmo polivalente propuesto por James Westgard y col., que se implementó en multitud de analizadores automáticos [3]. En aquel momento, todas las aplicaciones del IQC se han definido como control estadístico de procesos (CEP), aunque no se tenía en cuenta la utilidad clínica de los resultados de la prueba para el diseño o planificación de las aplicaciones de dicho CEP. El desarrollo del concepto de “error analítico total aceptable” y la búsqueda de requisitos de calidad que proporcionaran una guía objetiva de evaluación u utilización de los métodos en control de la calidad, dieron lugar a que el IQC se abordara desde un punto de vista más clínico. Finalmente, en la Conferencia de Consenso de Estocolmo de 1999 [4] y la I Conferencia Estratégica de la Federación Europea de Medicina de Laboratorio (EFLM) [5] celebrada en Milán, se estableció una jerarquía clara de modelos para establecer especificaciones de la calidad no solo para los errores aleatorios de control, sino para que los laboratorios clínicos pudieran comprobar la alineación del sistema con las referencias de orden superior [6, 7]. El artículo reconoce los enormes esfuerzos realizados por algunos investigadores eminentes del campo de la medicina de laboratorio, así como el papel de las sociedades científicas y federaciones, incluida la Sociedad Española de Medicina de Laboratorio (SEQC ^ML^), que tuvo una gran implicación en las iniciativas internacionales para mejorar el IQC y la EQA. Así mismo, se ha logrado realizar importantes avances en la “naturaleza” de los materiales de control, como su suministro por parte “de terceros” y, lo que es más importante, su conmutabilidad con los resultados obtenidos en el suero del paciente. No obstante, este escenario sigue en constante evolución, tal como demuestran dos estudios realizados en 2017 y 2021 respectivamente, en los que se analizan protocolos de IQC, citados en el artículo. Lamentablemente, los resultados de los dos estudios revelan que existe un conocimiento escaso por parte los laboratorios clínicos sobre los requisitos de IQC actualmente reconocidos, incluyendo los materiales de control proporcionados por terceros, el uso de normas de control operativo obsoletas, o la baja valoración de la importancia de que el sistema esté alineado con una referencia según unos criterios de trazabilidad metrológica. Además, un editorial publicado recientemente en *in Clin Chem Lab Med* [8], en el que se comentan dos artículos de James Westgard [9] y Mauro Panteghini [10], pone de manifiesto la necesidad de intensificar esfuerzos para que los profesionales de laboratorio adopten programas de IQC actualizados y más precisos. Además, el artículo de Carmen Ricós y col. hace hincapié en el debate existente sobre el control de media móvil y, aún más importante, el control de calidad centrado en el paciente, que deberían complementar y ser incorporados a los programas de IQC tradicionales [11, 12]. Quisiéramos recordar que la espectacular reducción del número de errores analíticos observada en las últimas décadas no solo se debe a la automatización y optimización de los instrumentos y metodologías, sino también a la adopción de programas de IQC y EQC, que han permitido mejorar la calidad analítica en el propio laboratorio, así como la evaluación comparativa entre laboratorios.

El segundo artículo publicado por Carmen Ricós y col [2]. complementa el anterior y resume la evolución de los distintos tipos de programas de garantía externa de la calidad, partiendo del primer programa “inventado” por Belk y Sunderman en 1947 [13] para evaluar ensayos de bioquímica clínica y el primer programa de hematología diseñado por Lewis y Burgess en 1969 [14]. Aunque el propósito de los programas de garantía externa de la calidad (EQA) y de evaluación de competencia (EC) están destinados a determinar la inexactitud e imprecisión de los resultados entre laboratorios clínicos, en Europa siempre se ha preferido utilizar esquemas de EQA, por poner mayor énfasis en su naturaleza formativa. Sin embargo, tal como comentan los autores, la convivencia de dos tipos de EQ (obligatorio/EC y formación) da lugar a que un laboratorio que participe en los dos tipos de programas reciba un informe de calidad aceptable para un analito, y otro de calidad inaceptable en el otro programa, tal como han señalado Badrick y Stavelin [15]. A la hora de definir la EQA, los autores explicaron la diferencia semántica entre los términos *“assessment”* (evaluación) y *“assurance”* (garantía), que a veces se utilizan indistintamente. Sin embargo, el segundo término se debería emplear para hacer hincapié en que “no se trata solo de una evaluación de la calidad analítica, sino que también se evalúa la interpretación de los resultados de las pruebas y las recomendaciones a los facultativos sobre el valor diagnóstico de los mismos” [12]. Los autores señalan los siguientes progresos en los programas de EQA; a) la naturaleza de los materiales de control, concretamente, su conmutabilidad y asignación de valor; b) el establecimiento de especificaciones de la calidad para la evaluación de resultados; c) la introducción de evaluaciones de calidad extra-analítica [16, 17]; d) y la armonización de los programas de EQA existentes. Concretamente, Miller y col identificaron los factores clave a la hora de evaluar la fortaleza de un programa EQA, estableciendo seis categorías [18]: a) las categorías 1 y 2 emplean materiales conmutables con valores asignados mediante métodos de referencia certificados; la categoría 1 verifica tanto la precisión como la reproducibilidad, mientras que la categoría 2 únicamente verifica la precisión; b) las categorías 3 y 4 emplean materiales conmutables, pero no asignan valores empleando materiales de referencia certificados; c) finalmente, las categorías 5 y 6 emplean materiales de control no conmutables sin valores asignados. Es evidente que los laboratorios clínicos deben tratar de adoptar programas de EQA de categoría 1 y 2, evitando participar en las categorías 5 y 6, mientras que se deberían lanzar iniciativas nacionales e internacionales para ayudar a los laboratorios a seleccionar estos programas, superando las barreras económicas y regionales.

El establecimiento de especificaciones de la calidad y la armonización de las especificaciones empleadas en los programas de EQA continúa siendo una pesadilla, aunque ya se han identificado los pilares para la estandarización/armonización, que los autores resumen de la siguiente manera: 1) materiales de referencia certificados; 2) procedimientos de medida de referencia; 3) laboratorios de referencia reconocidos; 4) intervalo de referencia acordado; 5) programas de EQA comparables.

Sin embargo, aún existe margen de mejora en los programas de EQA mediante el desarrollo y puesta a disposición de procedimientos de medida de referencia y materiales de referencia certificados (o reconocidos internacionalmente). De otro modo, solo existirían programas de EQA normalizados con trazabilidad metrológica para 25 o 30 analitos. Además, es necesario alcanzar un consenso para definir y mejorar la calidad de los programas de EQA para todos los analitos.

En resumen, los dos artículos publicados en este número de la revista no solo ofrecen la oportunidad de actualizar nuestros conocimientos sobre la historia del IQC y la EQA, sino que también nos permitirán repensar e identificar los aspectos que quedan por resolver y que deberán ser abordados mediante iniciativas de mejora en un futuro próximo.
